# Delayed Hemolytic Transfusion Reaction With Hyperhemolysis Syndrome Due to Anti-M Alloantibody in Myelofibrosis: A Case Report

**DOI:** 10.7759/cureus.50717

**Published:** 2023-12-18

**Authors:** Mohammad S Alsoreeky, Laith K Lutfi, Ahmad A Altamimi, Tamer H Haddad, Mashael S Khalayleh, Mohammad S Alkader

**Affiliations:** 1 Department of Clinical Oncology, Jordanian Royal Medical Services, Amman, JOR; 2 Department of Medical Oncology, Jordanian Royal Medical Services, Amman, JOR; 3 Department of Radiotherapy, Jordanian Royal Medical Services, Amman, JOR; 4 Department of Pathology, Jordanian Royal Medical Services, Amman, JOR

**Keywords:** anti-m alloantibody, steroid, splenectomy, myelofibrosis, dhtr, hyperhemolysis syndrome

## Abstract

Hyperhemolysis syndrome (HHS) and delayed hemolytic transfusion reaction (DHTR) commonly occur in patients with sickle cell disease (SCD) and thalassemia, due to the need for recurrent red blood cell (RBC) transfusion, but rarely in patients with myelofibrosis. HHS is a life-threatening condition that occurs with or without DHTR, in which both transfused and autologous RBCs are destroyed. It needs a high clinical suspicion for diagnosis, especially when there is a drop in hemoglobin level to the level of pretransfusion of RBCs, accompanied by hyperbilirubinemia and reticulocytopenia. The management of HHS includes avoiding RBC transfusion, supportive care, and immunomodulatory therapy. We present a case of HHS with DHTR in a patient with primary myelofibrosis who was treated successfully with steroids and splenectomy.

## Introduction

Primary myelofibrosis (PMF) is a type of myeloproliferative disorder. It is characterized by megakaryocytic proliferation, bone marrow fibrosis, anemia, and splenomegaly [1,2]. The disease is caused by driven mutations in Janus Kinase-2 (JAK2), myeloproliferative virus (MPL), or calreticulin (CALR) [3].

A delayed hematological transfusion reaction (DHTR) is a hematological reaction that occurs a few days to weeks post-RBC transfusion due to alloimmunization [4]. Hyperhemolysis syndrome (HHS) is a life-threatening condition occurring post-RBC transfusion, commonly seen in sickle cell anemia, b-thalassemia, and rarely in myelofibrosis [5].

The diagnosis of HHS is difficult and needs high clinical suspicion, especially after a drop in hemoglobin level post-RBC transfusion to the level of pre-transfusion, accompanied by reticulocytopenia [6]. There are two categories of HHS, the first one is acute type which occurs in the first seven days post transfusion, and the second one is a delayed type which occurs after seven days of transfusion [7].

The treatment of HHS is primarily to decrease hemolysis by using steroids and intravenous immunoglobulin (IVIG). However, in refractory cases, treatment includes anti-C5 (eculizumab), anti-CD20 (rituximab), and plasma exchange, and along with other supporting products to enhance erythropoiesis, includes erythropoietin (EPO), intravenous (IV) iron, vitamin B12, and folate [8].

## Case presentation

A 40-year-old male patient known to have beta thalassemia trait and primary myelofibrosis with intermediate-2 risk since March 2019, was started on Jakavi (roluxitinb). After three years of Jakavi, in December 2022, he complained of abdominal pain, easy fatigability, and general weakness. On physical examination, the patient showed a pallor and had splenomegaly below the umbilicus. Laboratory investigations showed a hemoglobin level of 6.2 g/dl, and abdominal ultrasound demonstrated huge splenomegaly.

The patient was given 2 units of packed red blood cells (pRBCs), and after seven days of transfusion, he complained of fever, chills, and red urine. Laboratory investigations revealed hemoglobin level dropped to 4.2 g/dl, bilirubin was 6.8 mg/dl, lactate dehydrogenase (LDH) was 1450 u/l (reference range: 100-248), direct antiglobulin test (DAT) IgM was positive +2, and the presence of anti-M alloantibodies was detected (Table 1).

**Table 1:** Initial laboratory investigation

Laboratory Investigation	Result
White blood cell	12.3 * 10^9^/L
Hemoglobin	4.2 g/dl
Mean corpuscular volume	85 fl
Platelet	95 * 10^3^/µL
Lactic dehydrogenase	1450 u/l
Bilirubin	6.8 mg/dl
Direct antiglobulin	IgM positive +2
Anti-M alloantibody	positive
Reticulocyte	2.7%

The patient was started on dexamethasone 8 mg twice daily with 1 unit of RBC phenotypically matched; his hemoglobin dropped to 3.5 g/dl, and reticulocyte count was 2.7%. Upon decreasing hemoglobin level with RBC transfusion to the level of pre-transfusion, high bilirubin, high LDH, and reticulocytopenia, the diagnosis of DHTR with HHS was suspected. After three days of dexamethasone, high dose methylprednisolone 1 gm daily for five days was initiated while stopping RBC transfusion. His hemoglobin level improved, reaching 6.1 g/dl without further improvement.

**Figure 1:**
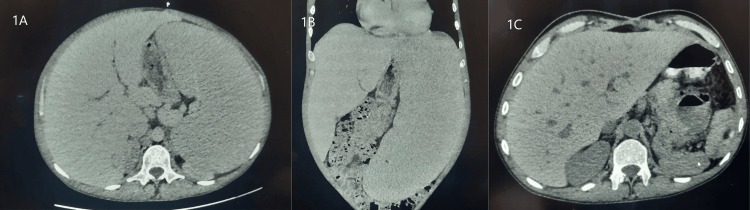
Huge splenomegaly in myelofibrosis patient A, B: axial and coronal view of abdominal CT scan shows huge splenomegaly; C: post splenectomy

**Figure 2:**
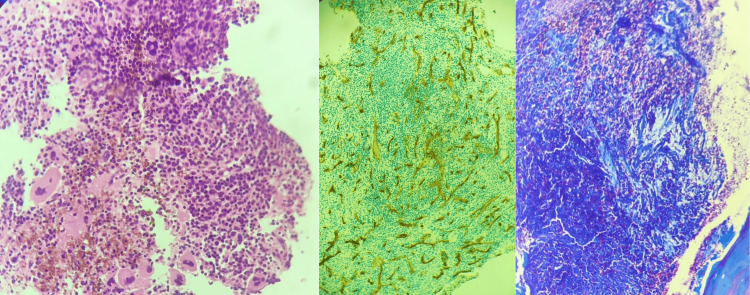
Bone marrow biopsy Megakaryocytes are increased with abnormal streaming pattern and cluster formation, reticulin stain shows grade 3 fibrosis, and Masson trichrome stain shows collagen fibrosis

The patient complained of left upper quadrant pain, and an abdominal CT scan showed huge splenomegaly (Figure 1A, 1B). Bone marrow biopsy was performed, revealing hypercellular bone marrow with areas replaced by fibrosis, megakaryocytes were increased with abnormal streaming pattern and cluster formation, reticulin stain showed grade 3 fibrosis, and Masson trichrome stain showed collagen fibrosis, without evidence of leukemic transformation (Figure 2).

**Figure 3:**
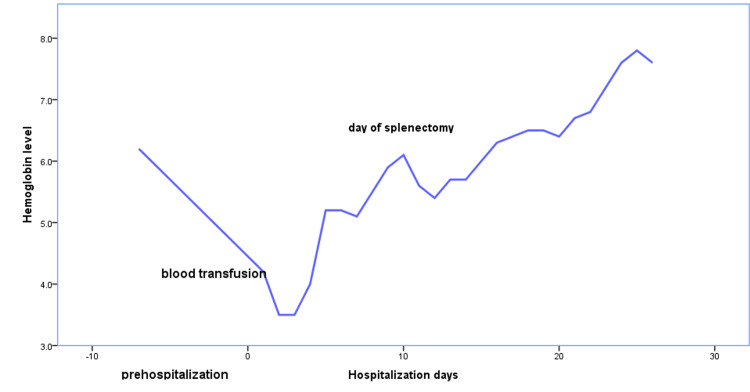
Timeline of hemoglobin level

The multidisciplinary team recommended splenectomy for this patient due to the risk of spleen rupture upon the discontinuation of Jakavi and progressive enlargement of the spleen. The procedure was performed successfully, and the patient's condition improved significantly (Figure 1C). Hemoglobin levels rose from 6.1 to 7.4 g/dl, and bilirubin returned to normal levels. He was discharged at this point in good general condition, and two weeks after discharge, his hemoglobin level reached 9.5 g/dl (Figure 3).

## Discussion

PMF is the most aggressive type of myeloproliferative neoplasms. Anemia, constitutional symptoms, and splenomegaly are the cardinal features, and generally, PMF is categorized into two subgroups: proliferative and cytopenic types [9,10].

HHS commonly occurs in sickle cell disease (SCD) and beta-thalassemia [5], but rarely occurs in other hematological disorders such as chronic lymphocytic leukemia, myelofibrosis, anemia of chronic disease, marginal zone lymphoma, and anemia of chronic disease [11].

HHS has two types: acute and delayed types. In the acute type, the hemolysis occurs within seven days of RBC transfusion, without new antibody formation against the transfused RBCs, with negative DAT, while the delayed type commonly occurs after seven days of RBC transfusion, accompanied by new antibody formation, and positive DAT [11]. In this case, the patient presents with a hemolytic reaction after eight days of RBC transfusion, with positive DAT and anti-M alloantibody detected.

The pathophysiology of HHS is not clear and there is no scientifically proven mechanism, but it may be related to hyperactivation of macrophage, suppression of erythropoiesis, and bystander hemolysis [6]. The patient in the current report had a huge splenomegaly, which may be a risk to developing HHS since splenic red pulp contains a large number of macrophages leading to RBC destruction [7]. The pathophysiology in the acute type may related to the hyperactivation of macrophage, due to the absence of alloantibody and negative DAT, while in the delayed type, it may be related to antibody-mediated hemolysis through the formation of new alloantibody, which destroys transfused RBCs, after which destruction of autologous RBCs may occur by recruitment of macrophage [6].

Splenectomy in patients with PMF is debatable. It has a disease-modifying effect that decreases the risk of graft failure and reduces relapse-risk mortality post-hematopoietic stem cell transplant, but it may increase the risk of infections and thrombosis [12]. Splenectomy was indicated to our patient due to symptomatic and progressive splenomegaly [13]; further improvement and rising of hemoglobin level and normalization of bilirubin post splenectomy were noticed. However, Rehman et al. reported unclear benefits of splenic embolization for HHS in patients with SCD [14].

Treatment of HHS include avoiding transfusion, except for life-saving condition, consideration of immunomodulatory therapy (steroid, IVIG, eculizumab, and rituximab), and supportive care [8]. Plasma exchange has been reported in the treatment of HHS, resulting in clinical improvement and rising hemoglobin levels [15]. This may relate to the removal of auto and alloantibodies, by the removal of which, the level of free plasma hemoglobin decreases. Once the free hemoglobin decreases, the hemolysis decreases.

Our patient was treated by avoiding further transfusion and high dose methylprednisolone, which led to an improvement of hemoglobin level reaching 6.1 g/dl; after that, he underwent splenectomy with further improvement in hemoglobin reading. Initially, the patient was treated by stopping further transfusion and steroids including dexamethasone and methylprednisolone without using other modalities such as rituximab, eculizumab, IVIg. Here, we can assume that the patient may have improved due to splenectomy.

## Conclusions

HHS rarely occurs in patients with myelofibrosis who receive RBC transfusions, and requires high clinical suspicion. An early and accurate diagnosis, followed by individualized interventions, such as splenectomy in certain situations, can result in better outcomes and emphasizes the significance of a comprehensive and personalized approach in the handling of intricate hematological disorders. The case adds to the small amount of knowledge about HHS in the context of myelofibrosis, and emphasizes the significance of ongoing research in this field to expand our understanding and enhance patient care.
